# Transvalvular Mechanical Circulatory Support Reverse Remodels the Ischemic Chronic Failing Heart in an Ovine Model

**DOI:** 10.1016/j.jacbts.2025.01.003

**Published:** 2025-03-19

**Authors:** Bart Meyns, Randi J. Parks, Erik Verbeken, Manon van Hecke, Steven Jacobs, Magdalena Sikole, Charlotte Van Edom, Eveline Bennek Schoepping, Piet Claus

**Affiliations:** a Department Cardiac Surgery, KULeuven, Leuven, Belgium; b Academic and Pre-Clinical Research, Abiomed, Inc, Aachen, Germany; c Department of Pathology, KULeuven, Leuven, Belgium; d Department of Cardiovascular Sciences, KULeuven, Leuven, Belgium; e Department of Cardiovascular Imaging, KULeuven, Leuven, Belgium

**Keywords:** chronic heart failure, mechanical circulatory support, MRI, myocardial infarction, ovine, reverse remodeling

## Abstract

Sixteen sheep with ischemic chronic heart failure were randomized to either 3 months of Impella 5.5 support (Abiomed) or sham. Myocardial reverse remodeling by Impella support was shown by a significant reduction of end-diastolic volume (Impella 22% reduction, and control 5% increase; *P =* 0.011) and end-systolic volume (Impella 20% reduction, and control 11% increase; *P =* 0.011). Functional measurements demonstrated improved regional ejection fraction and wall thickening in the viable myocardium of hearts supported with Impella. Supported animals had a significant decrease in wall volume, with less cardiomyocyte hypertrophy than control subjects.

## Highlights

• Mechanical support reversely remodels the chronic failing heart.
• Transvalvular support with Impella is less invasive and does not require coring of the apex.
• Three months of Impella support reversely remodels the ischemic chronic failing heart in an ovine model and improved contractility in the remote zone.

## Visual Abstract

**Figure:**
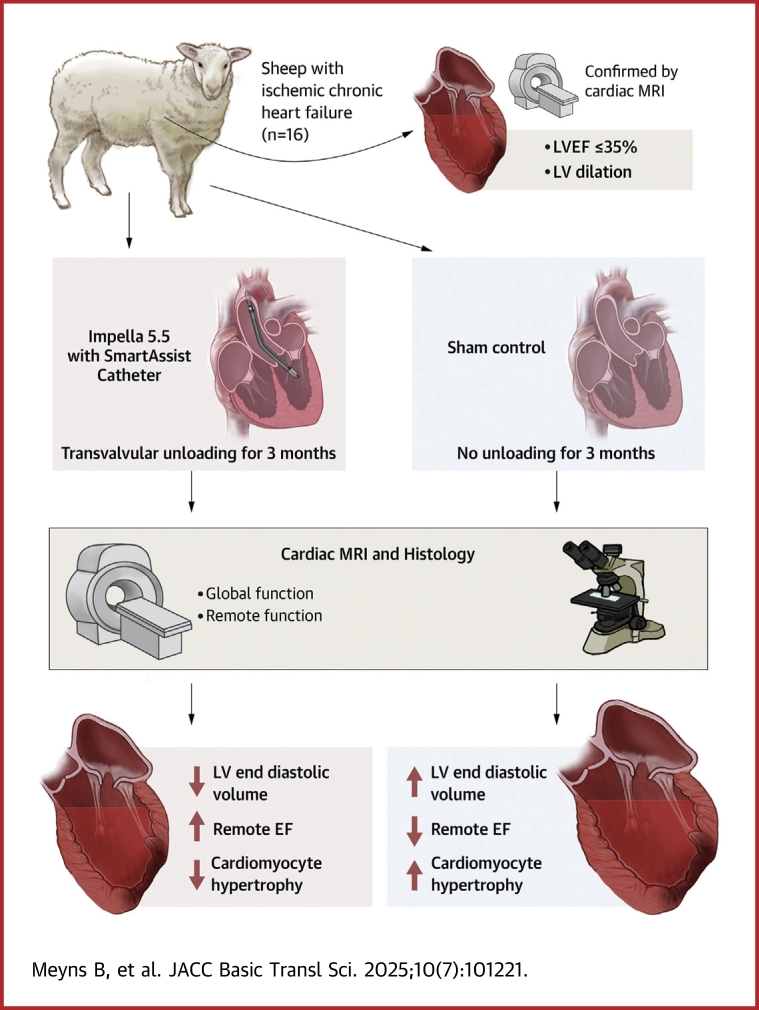

Prolonged hemodynamic overload leads to adaptive structural and molecular changes in the myocardium, including alterations in the size, shape, and composition of the ventricles. As chronic heart failure (CHF) progresses, the continued pressure and volume overload pushes the heart to remodel beyond a physiological state, yielding a pathologically remodeled myocardium with altered function.^1^^,^^2^

Recovery from CHF is broadly defined as reversal of the pathological state with significant improvement in cardiac structure and function sufficient to achieve a sustained remission from recurrent heart failure (HF) events.^3^ Heart failure therapies, therefore, should act to halt further pathological remodeling and, ideally, reverse the remodeling that has occurred over the duration of the disease. Reverse remodeling results in a new, less pathological state that is more robust but still fragile and susceptible to future cardiac events.^1^^,^^4^ Depending on the degree of reverse remodeling, patients may achieve a remission from HF with extended periods without clinical events but susceptible to future cardiac events or a complete recovery from CHF with sufficient reverse remodeling leading to freedom from future cardiac events.^1^ Although complete recovery is the ideal treatment outcome, remission from CHF is clinically meaningful, with patients experiencing years without worsening symptoms or rehospitalization. However, even with the tools and technology available today, achieving remission or recovery of CHF is challenging.

Among current therapies, the therapeutic modality that has shown the most promise in promoting HF remission and recovery is ventricular unloading device therapy, such as left ventricular assist devices (LVADs) that directly unload the overloaded left ventricle (LV) and provide forward flow. In CHF patients supported with LVAD, reversal from CHF has been reported with varying incidence from 1% to 11%, with the highest incidence of 50%, depending on the patient population and clinical strategy employed.^5^ The RESTAGE-HF (Remission from Stage D Heart Failure) trial showed that with a narrow population of young patients with nonischemic cardiomyopathy, LV ejection fraction (EF) <25%, and HF duration ≤5 years, recovery to device explantation was achieved in 50% of the study subjects when supported with HeartMate II LVAD (Abbott) and guideline-directed medical therapy (GDMT) for 18 months.^6^ To fully harness the possibility of recovery, it is imperative to treat patients at the right time when they still have the potential to significantly reverse remodel. The REVIVE-IT (Randomized Evaluation of VAD InterVEntion before Inotropic Therapy) trial aimed to determine whether LVAD therapy in less advanced HF patients than currently indicated for LVAD (NYHA functional class IV or advanced III not dependent on inotropes; 72% were INTERMACS 6 or 7) would improve clinical outcomes.^7^ REVIVE-IT compared optimal medical management with permanent LVAD use in the less sick population, but encountered a lack of equipoise because of the concern of exposing these less sick patients to increased risk, in particular pump thrombosis and stroke. Furthermore, LVADs require coring of the apex, removing a functional portion of the heart. This invasive procedure could contribute to development of fibrosis and inflammation.

Treatment options with a better benefit-to-risk ratio are needed to allow for the recovery strategy to be considered as a therapeutic option for CHF patients. A less invasive mechanical circulatory support (MCS) device providing active LV unloading with sufficient forward flow could optimize myocardial reverse remodeling without damaging the native heart tissue. This study aimed to investigate the hypothesis that ventricular unloading with a transvalvular mechanical circulatory support system, Impella 5.5 (Abiomed), would halt adverse remodeling in an ovine model of ischemic HF and promote reverse remodeling. This study is a proof-of-concept for a less invasive transvalvular mechanical support device to facilitate reverse remodeling of CHF.

## Methods

### Animal preparation and management

The procedures performed were approved by the ethics committee of KU Leuven. All animals were monitored by a veterinarian, according to the “Guide for the Care and Use of Laboratory Animals’’ published by the National Institutes of Health.

Swifter female sheep (approximately 50 kg and age 1.5-2 years) were obtained from the Zootechnical Center of KU Leuven and housed at the animal facility before surgery. For procedures, animals were sedated (10-20 mg/kg ketamine intravenous), induced with 5% isoflurane via face mask, intubated, and mechanically ventilated. Anesthesia was maintained with isoflurane (2.0%-2.5%). A peripheral venous line and deep central catheter were placed in the left saphenous and jugular vein, respectively. Peripheral arterial pressure was measured invasively in an ear artery via a fluid-filled line connected to a pressure transducer (Maquet GmbH). A large 23-F gastric tube was placed in the stomach to prevent ruminal distension. Pulse oximetry and electrocardiogram monitoring were placed on the animal.

Prophylactic antibiotics (penicillin 12 mg/kg and gentamycin 6.6 mg/kg) and pain medication (Metacam [Boehringer Ingelheim] 0.5 mg/kg and buprenorphine 0.1 mg/kg) were given intramuscularly before and after surgical procedures, with a 24-hour frequency of dosing.

### Coronary ligation

A 5,000 IU heparin loading dose was given before coronary ligation to prevent clot formation and stroke. To reduce the incidence of arrhythmias, an infusion of 300 mg amiodarone in 5% glucose and 250 mg lidocaine in 200-mL 0.9% NaCl was initiated before infarct induction to achieve a prophylactic concentration.

**Figure 1:**
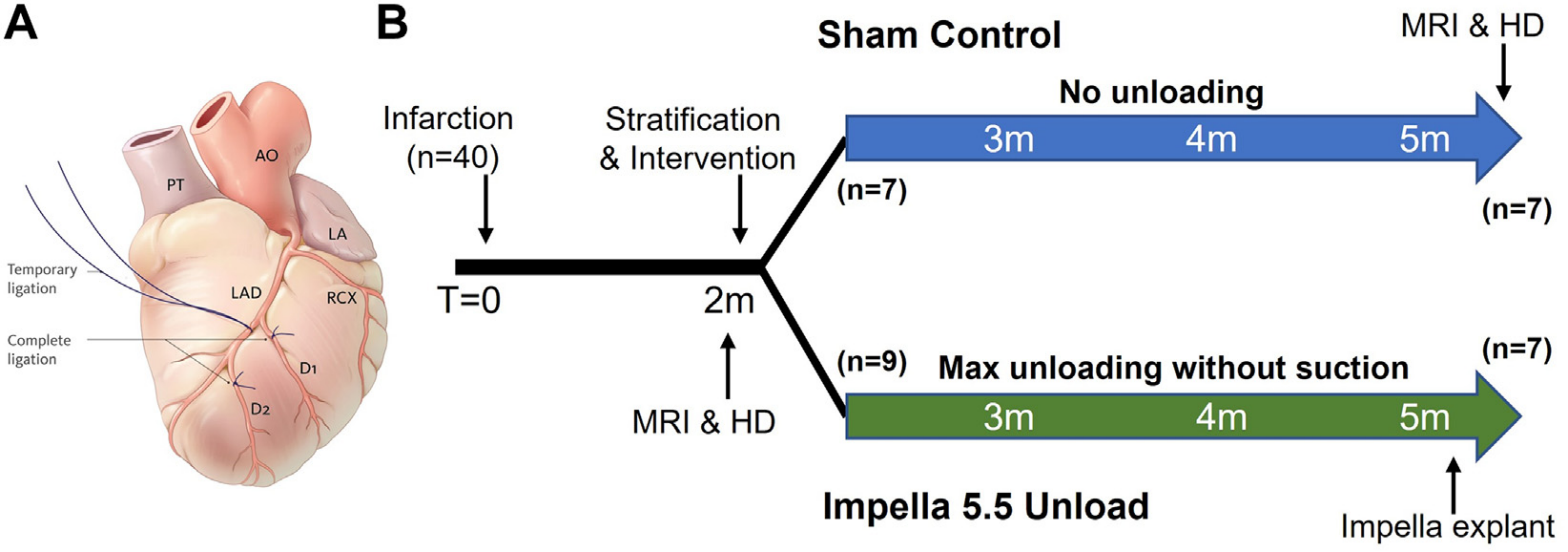
Ovine Animal Protocol Timeline (A) Following infarction of the mid-left anterior descending artery (LAD) and diagonals (D1, D2), (B) sheep were monitored for 2 months while heart failure developed, at which point they were stratified to receive Impella 5.5 or sham control. After 3 months of unloading or control, necropsy was performed. Magnetic resonance imaging (MRI) and hemodynamics (HD) were assessed at various timepoints. AO = aorta; LA = left atrium; PT = pulmonary trunk; RCX = ramus circumflex.

A combination of permanent ligation and ischemia/reperfusion was performed in series to obtain a realistic clinical substrate. The ligation was done in an open chest model and combines 90 minutes of ischemia followed by reperfusion of the mid-left anterior descending artery, with subsequent permanent ligation of the diagonal branches (Figure 1A).^8^ Regional ischemia was achieved by snaring the coronary with a 3-0 polypropylene suture and confirmed by visualizing cyanosis, loss of regional contractility, and the occurrence of typical electrocardiogram changes of ischemia.

All sheep developed low cardiac output (CO) and were treated with noradrenaline to maintain mean arterial pressure >40 mm Hg. A bolus injection of MgSO_4_ (2 g) was given immediately after coronary occlusion.^8^ Typically, 30 to 40 minutes after coronary occlusion, ventricular fibrillation occurred and was treated by defibrillation and resuscitation if necessary. The mortality of the CHF model is significant (50%). Cause of death was predominantly ventricular fibrillation in the first hours and days after myocardial infarction.

### Impella 5.5 implant and sham control

CHF developed over 2 months, at which point animals underwent cardiac magnetic resonance (CMR) to assess their EF and LV dilation. Animals with LVEF ≤35% and LV dilation were deemed in CHF and were stratified to receive Impella 5.5 implant or sham surgery (Figure 1B). Sham surgery consisted of thoracotomy without placement of an Impella. A subset of animals that did not meet the criteria were reinfarcted and monitored for another 2 months before intervention (n = 2 control and 3 Impella).

Anesthesia was performed as described in the previous text. A left lateral thoracotomy at the fourth intercostal space was made. For pump insertion, a beveled end-to-side anastomosis was created between an 8-mm knitted graft and the ascending aorta, at least 4 cm above the aortic valve. Then, Impella 5.5 was inserted over a 0.018-inch guide wire, with the inflow in the LV and the outflow in the ascending aorta. Pump position was verified by X-ray and the pump pressure signals, and silicone plugs were used to maintain the pump position. Pump speed was adjusted to the highest possible flow without suction. After Impella implant or sham procedure, sheep were monitored for 3 months with continued support. At 3 months postintervention (which is 5 months postinfarct), the Impella pumps were removed, invasive hemodynamics and CMR were performed, and all animals were sacrificed.

### Magnetic resonance imaging

At 2 months postinfarct before randomization and 3 months postintervention after Impella explant, CMR was performed under general anesthesia. In the Impella supported animals, the CMR was performed following the surgery to remove the pump during the same anesthesia (within 2 hours of pump removal). Imaging was performed on a 3-T magnet (Magnetom Prisma^fit^, Siemens Healthineers). Animals were installed in right decubitus position and ventilated throughout imaging. In addition to the spine coil, a phased-array flex coil was wrapped around the chest for better signal-to-noise ratio. Images were acquired with electrocardiogram-gating where appropriate and under suspended respiration (end-expiratory/open mouth).

Cine images were acquired in 6 long-axis slices centered and equally distributed (every 30°) around the LV central axis (line connecting the apex and the midpoint of the mitral ring), in addition to a contiguous stack of short axes covering the complete left atrium and ventricle (±20 slices) and planned perpendicular to the former. All cine imaging was performed using a segmented 2-dimensional balanced steady state free precession sequence with a slice thickness of 6 mm and aiming at an in-plane pixel size of 1.3 × 1.3 mm (adapting matrix size and field-of-view to the sheep), using retrospective gating to reconstruct 30 phases per cardiac cycle. Typical sequence parameters were: echo time: 1.58 ms; repetition time: 39.71 ms; flip angle: 50°.

**Figure 2:**
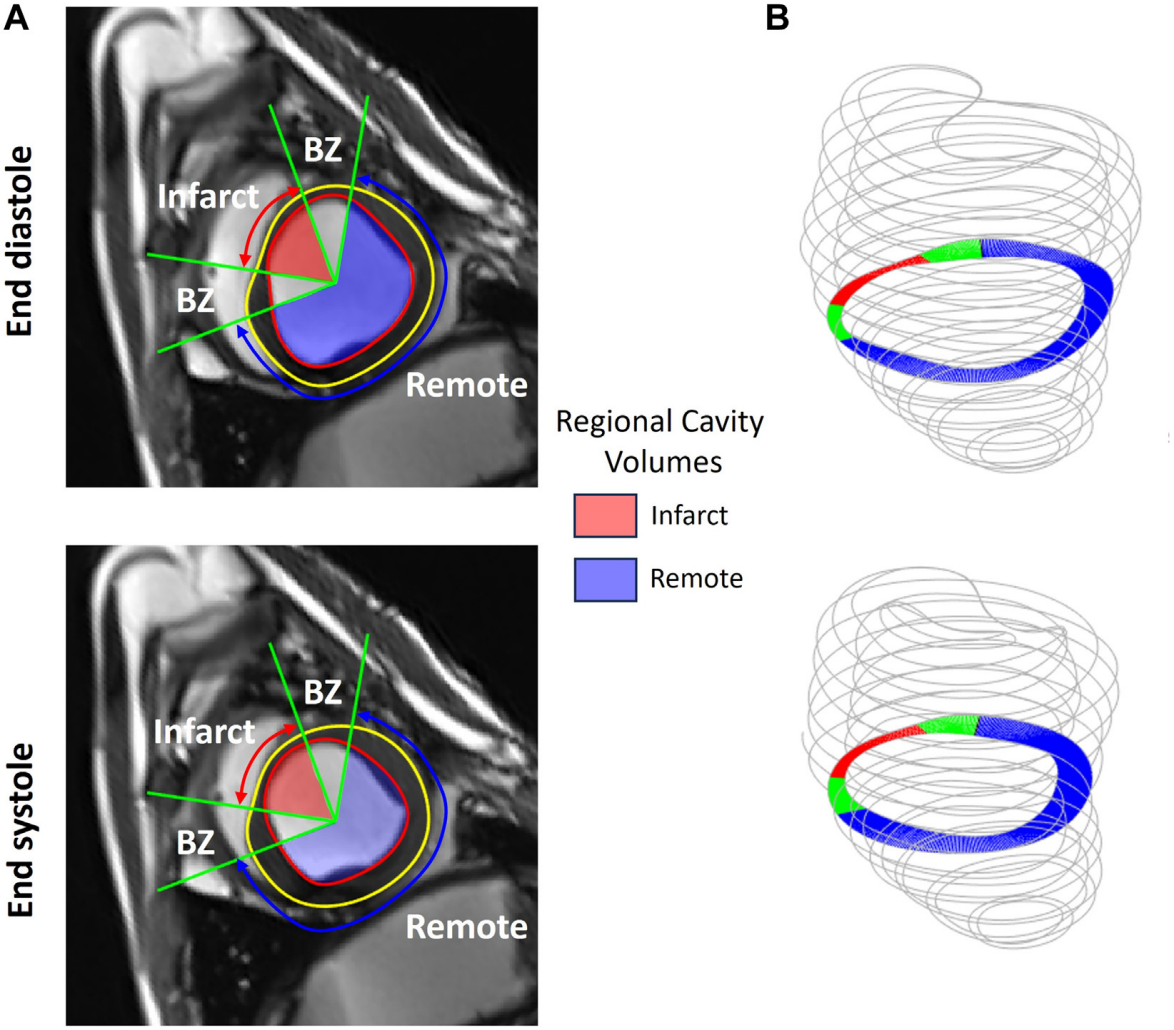
Cardiac Magnetic Resonance 3-Compartment Model and Regional Analysis (A) On each short-axis slice, 3 substrates are defined with infarct, border zone (BZ), and remote segments (when covered by that slice). Regional quantities can be analyzed, such as regional cavity volumes, defined as the volume subtending a certain substrate and other geometrical wall properties, such as substrate mass, wall thickening, and circumferential strain. The BZ is defined as a fix-sized (by angles) region adjacent to infarcted segments to limit including tethering effects between substrates. (B) Final quantities are obtained by summing all slices per substrate.

Endocardial and epicardial contours were manually drawn on end-diastolic and -systolic short-axis images—co-visualizing long-axis images for additional control—using dedicated software (RightVol, KULeuven), from which end-diastolic and -systolic LV cavity volumes and end-diastolic myocardial volumes were calculated using the summation of disks method.^9^ These parameters were used to calculate stroke volume (SV), EF, and CO. The infarcted myocardium was defined as thin-walled and/or akinetic segments. Infarct size was defined as percent infarcted myocardium relative to total myocardial volume. A 3-compartment model^10^ defining infarct, adjacent, and remote myocardium was adopted for regional measurements, such as regional myocardial volume, regional EF, circumferential strain, and wall thickening (Figure 2).

### Hemodynamic measurements

LV pressure tracings were made at 2 and 5 months postinfarct immediately before CMR. A pressure guidewire (OptoWire III, OPSens) was calibrated at room air, inserted into the lumen of a pigtail catheter, and advanced into the LV under fluoroscopic guidance. LV pressure signals were recorded at 250 Hz. To account for respiratory variability under mechanical ventilation, cardiac cycles at end expiration were used.

End-diastolic and -systolic global and regional wall stress were calculated based on the Arts’ formula^11^: $\sigma =LVP\times \left(1+3\frac{cavity\;volume}{wall\;volume}\right)$, where wall volume is the total volume for global stress and the regional remote volume for regional stress.

### Necropsy

After the final CMR at 5 months postinfarct, sheep received heparin (3 mg/kg) intravenously and were sacrificed with an overdose of Euthasol solution intravenously. The heart was excised and opened along the lateral wall of the LV to allow for detailed evaluation of the LV with the induced infarction. For each animal, a biopsy of the infarcted myocardium, as well as a biopsy of the visually unaffected ventricular wall remotely located from the infarction was taken and stored immediately in 4% buffered formalin until further processing.

### Histology

After fixation, all biopsies were embedded individually in paraffin, after which 5-μm thick sections were provided of each paraffin block. A section of each biopsy was stained with hematoxylin and eosin (3801540BBE and 3801590BBE, Leica Biosystems). Using light microscopy, the histologic integrity of the tissue, the presence of an inflammatory response in the tissue, and the extent of the healing process were evaluated blindly by a pathologist.

Besides a morphologic assessment, an automated morphometric measurement of the remote myocardium of each animal was performed blindly by a dedicated pathologist to evaluate cardiomyocyte hypertrophy. The hematoxylin and eosin slide was first digitalized (Zeiss Axio Scan.Z1, Oberkochten) and subsequently analyzed using software (QuPath, version 0.3.2)^12^ to count the number of cell nuclei in a square of exactly 50,000 μm^2^ in 5 different histologically unremarkable areas, and means were taken. This mean count correlates inversely to volume of the individual cardiomyocytes, therefore lower values indicate hypertrophy.^13^

### Statistical analysis

Continuous variables are presented as mean and SD. Differences in cardiac volumes and hemodynamics were analyzed with a parametric 2-way repeated-measures analysis of variance to evaluate the time by treatment interaction. Normal distribution was confirmed with a Shapiro-Wilk test. Statistical significance was considered as *P <* 0.05. GraphPad Prism version 10.1.2 (GraphPad Software) was used for all analyses. The direct histological comparison of myocyte counts between hearts of control animals and supported animals was done with an unpaired Student’s t-test. Figures are presented using the data points with boxes and whisker plots to display the median, 25th-75th percentiles, minimum, and maximum values.

## Results

### Mortality and pump performance

Nine animals were supported with Impella 5.5, and 7 animals were treated as sham controls. Of the supported animals, 1 died from bleeding at the Impella access site overnight postimplant, and one was excluded because of frequent unmonitored suction events resulting in inadequate hemodynamic support. Impella 5.5 pump flow was manually adjusted to achieve maximum unloading without suction. Pumps were set between P6 and 7, with a mean flow rate of 4.0 ± 0.6 L/min. Three of the Impella devices required an intervention during pump support: 1 caused by animal interference, 1 caused by pump stop, and 1 caused by pump failure after rising purge pressure. At necropsy, assessment of the aortic wall, aortic valve, and endocardium showed no lesions or damage caused by the chronic presence of the pump in the Impella-supported animals.

### Global cardiac function and dimensions

**Figure 3:**
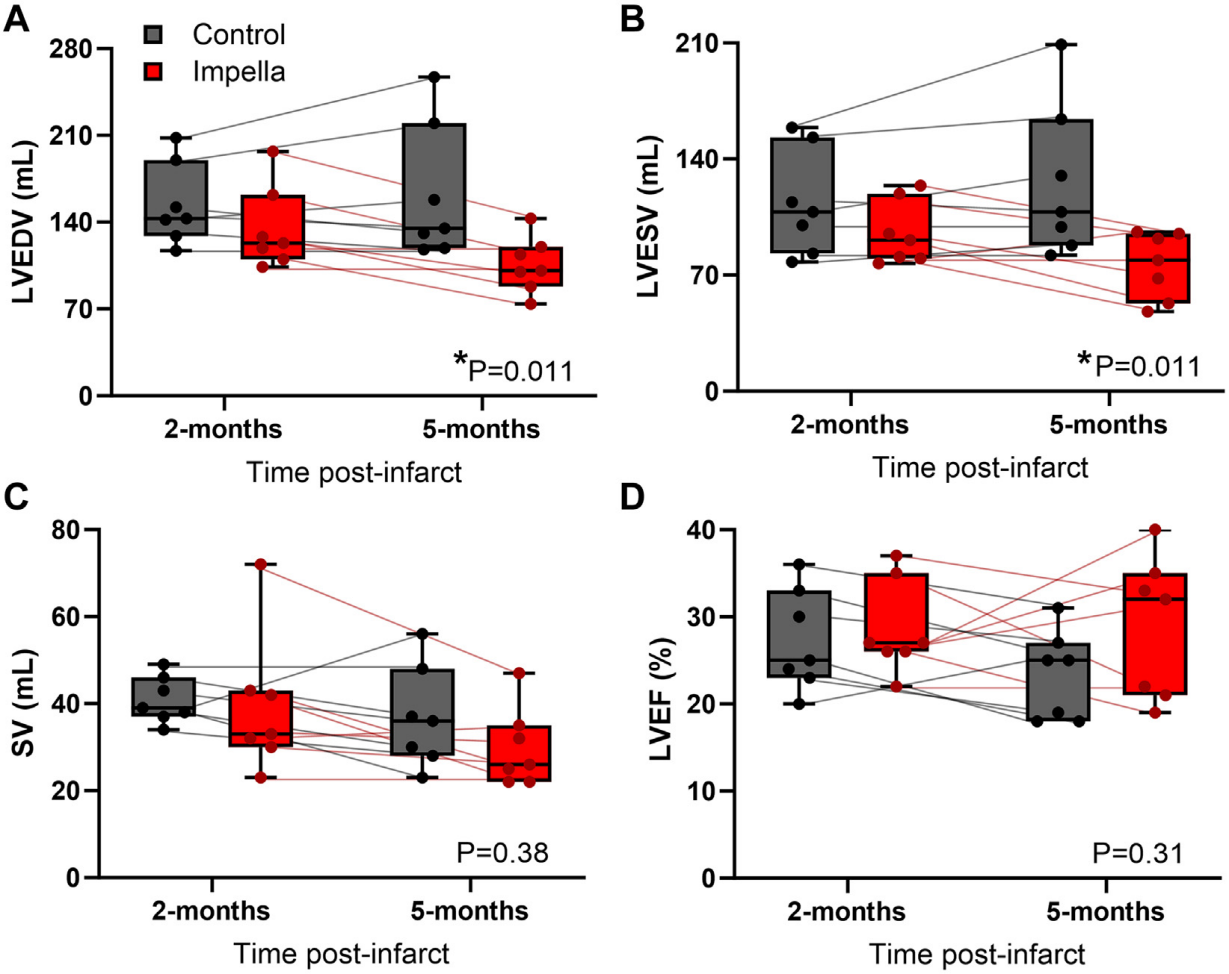
Transvalvular Unloading Improves Left Ventricular Volumes in Chronic Heart Failure Left ventricular volumes and function measured with cardiac magnetic resonance imaging at 2 months postinfarct and 3 months postintervention. Impella-treated hearts showed significantly reduced (A) left ventricular end-diastolic volume (LVEDV) and (B) left ventricular end-systolic volume (LVESV) in comparison to control, without altering the (C) stroke volume (SV) or (D) left ventricular ejection fraction (LVEF). Box plots show the median with 25-75th percentiles (n = 7 control, 7 Impella) ∗*P <* 0.05, 2-way repeated measures analysis of variance time × treatment interaction. Dots and thin lines represent individual animals for the control (black) and Impella (red) groups over time.

Following 3 months of treatment, CHF sheep after Impella support had significantly reduced LV end-diastolic volume (EDV) (Impella 2 months 135 ± 33 mL to 5 months 106 ± 22 mL; control 2 months 154 ± 33 mL to 5 months 163 ± 55 mL; *P =* 0.011) and end-systolic volume (ESV) (Impella 2 months 95 ± 19 mL to 5 months 76 ± 20 mL; control 2 months 114 ± 32 mL to 5 months 126 ± 46 mL; *P =* 0.011) in comparison to control (Figure 3). SV and EF were not significantly different between the 2 groups. CO was also unchanged between the Impella-treated and control animals (Impella 2 months 3.6 ± 1.1 L/min to 5 months 2.9 ± 0.9 L/min; control 2 months 3.7 ± 0.9 L/min to 5 months 3.1 ± 0.6 L/min; *P =* 0.843), while an improvement in LV sphericity index with Impella was not significant (Impella 2 months 0.51 ± 0.15 to 5 months 0.40 ± 0.07, and control 2 months 0.50 ± 0.09 to 5 months 0.48 ± 0.14; *P =* 0.134).

### Myocardial necrosis and scarring

Infarct size was similar between the control and Impella groups at 2 months postinfarct (13.4 ± 5.5% vs 9.1 ± 5.3% relative wall volume; *P =* 0.16) and 5 months postinfarct (13.3 ± 6.5% vs 10.0 ± 5.6% relative wall volume; *P =* 0.33). Total heart weight did not differ between the control and Impella animals at the time of necropsy (327 ± 39 g vs 388 ± 99 g, respectively; *P =* 0.22). LV weight (243 ± 35 g vs 209 ± 82 g; *P =* 0.45) and right ventricular weight (71 ± 23 g vs 92 ± 60 g; *P =* 0.51) were also unchanged between the 2 groups. Morphologically, scars of both study groups show the same features. The scars consist of an intact, transmural replacement of the myocardium by fibrofatty tissue without obvious inflammation, indicating the fully healed status of the infarction.

### Hemodynamics measurements

To determine the effect of unloading on LV wall stress, LV pressure was measured at 2 months postinfarct and 3 months after unloading or control. The hemodynamic findings are summarized in Table 1. Heart rate, mean arterial pressure, and CO were similar in both groups. There was a trend toward lower LV end-systolic pressure after 3 months of unloading (60.6 ± 16.2 mm Hg vs 84.7 ± 12.1 mm Hg).

**Table 1:** Hemodynamics and LV function After 3 Months of Transvalvular Unloading in Chronic Heart Failure

	Baseline		2 mo Postinfarct		5 mo Postinfarct		*P* Value
	Control	Impella	Control	Impella	Control	Impella	
Heart rate, beats/min	86 ± 14	90 ± 27	89 ± 19	95 ± 18	84 ± 18	99 ± 20	0.49
MAP, mm Hg	61 ± 18	58 ± 11	55 ± 12	57 ± 17	55 ± 11^1^	59 ± 11	0.77
CO, L/min	2.8 ± 0.3	2.4 ± 0.4	3.3 ± 0.7	3.2 ± 1.3	2.7 ± 0.3	2.7 ± 0.7	0.82
SV, mL	37 ± 6	34 ± 4	41 ± 5	39 ± 16	37 ± 12	30 ± 9	0.38
LVESP, mm Hg	61 ± 19	59 ± 13	69 ± 18	73 ± 12	85 ± 12	61 ± 16	0.079
LVEDP, mm Hg	11.6 ± 6.1	8.9 ± 1.5	9.7 ± 7.1	8.8 ± 5.8	12.8 ± 12.7	6.3 ± 4.1	0.59
LVESV, mL	55 ± 6	53 ± 18	114 ± 32	95 ± 19	126 ± 46	76 ± 20	0.023^a^
LVEDV, mL	92 ± 7	88 ± 20	154 ± 33	135 ± 33	163 ± 55	106 ± 23	0.020^a^
LVEF, %	40 ± 6	40 ± 6	27 ± 6	29 ± 5	23 ± 5	29 ± 8	0.44

Hemodynamic parameters in sham control and Impella-supported animals at baseline, 2 months postinfarct (before sham or Impella), and 5 months postinfarct (3 months after sham or Impella). Mean ± SD.

CO = cardiac output; LV = left ventricle/ventricular; LVEDP = left ventricular end-diastolic pressure; LVEDV = left ventricular end-diastolic volume; LVEF = left ventricular ejection fraction; LVESP = left ventricular end-systolic pressure; LVESV= left ventricular end-systolic volume; MAP = mean arterial pressure; SV = stroke volume.

a *P <* 0.05, 2-way repeated measures analysis of variance time × treatment interaction.

### Reverse remodeling in the remote zone

**Figure 4:**
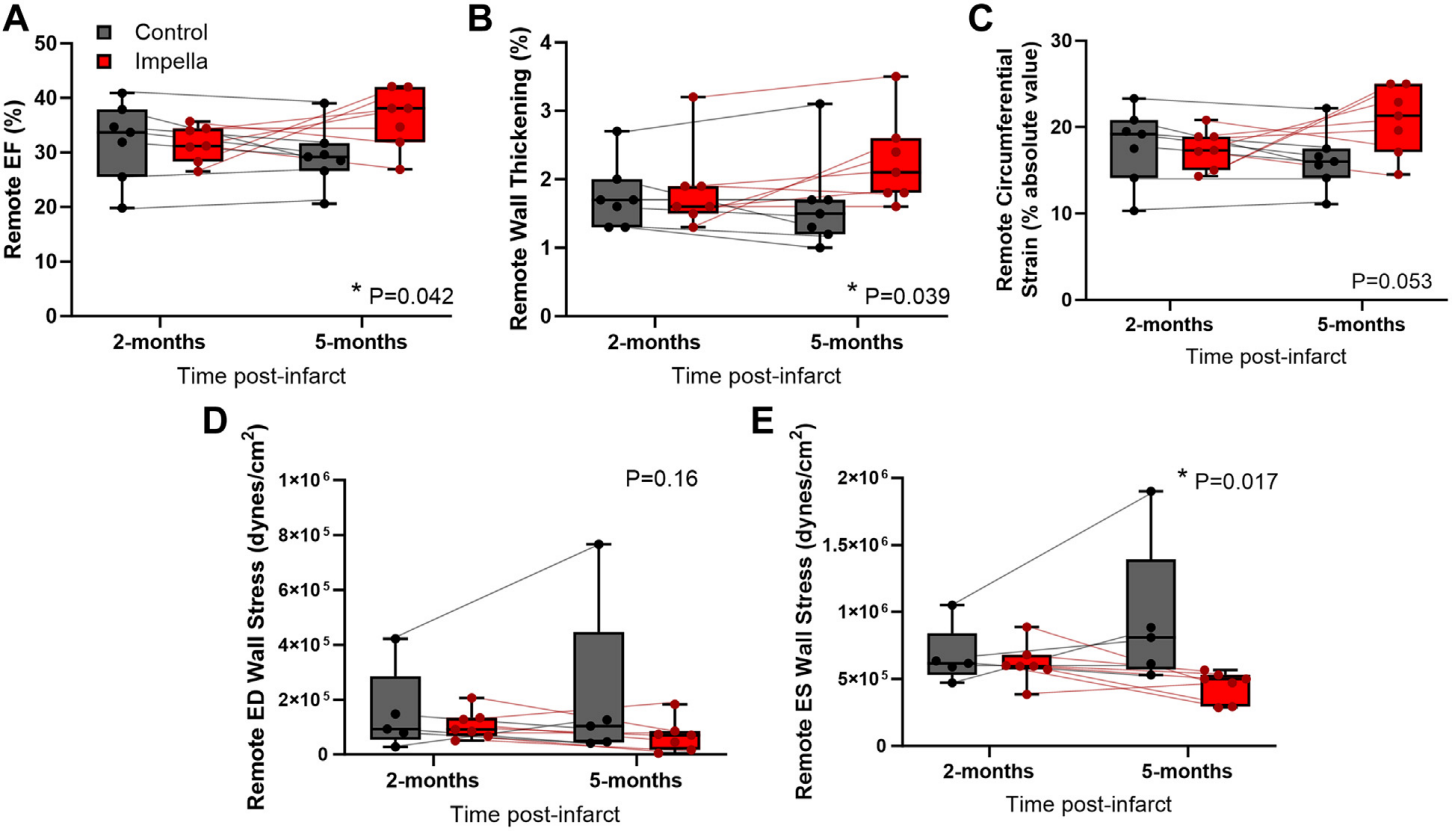
Transvalvular Unloading Improves Remote Function and Structure in CHF Regional functional measurements demonstrate a benefit of Impella on (A) regional ejection fraction (EF), (B) wall thickening, (C) circumferential end-systolic strain, (D) end-diastolic (ED) wall stress, and (E) end-systolic (ES) wall stress in the remote zone in the control and Impella groups. Box plots show the median with 25-75th percentiles (n = 7 control, 7 Impella) ∗*P <* 0.05, 2-way repeated measures analysis of variance time × treatment interaction. Dots and thin lines represent individual animals for the control (black) and Impella (red) groups over time.

**Figure 5:**
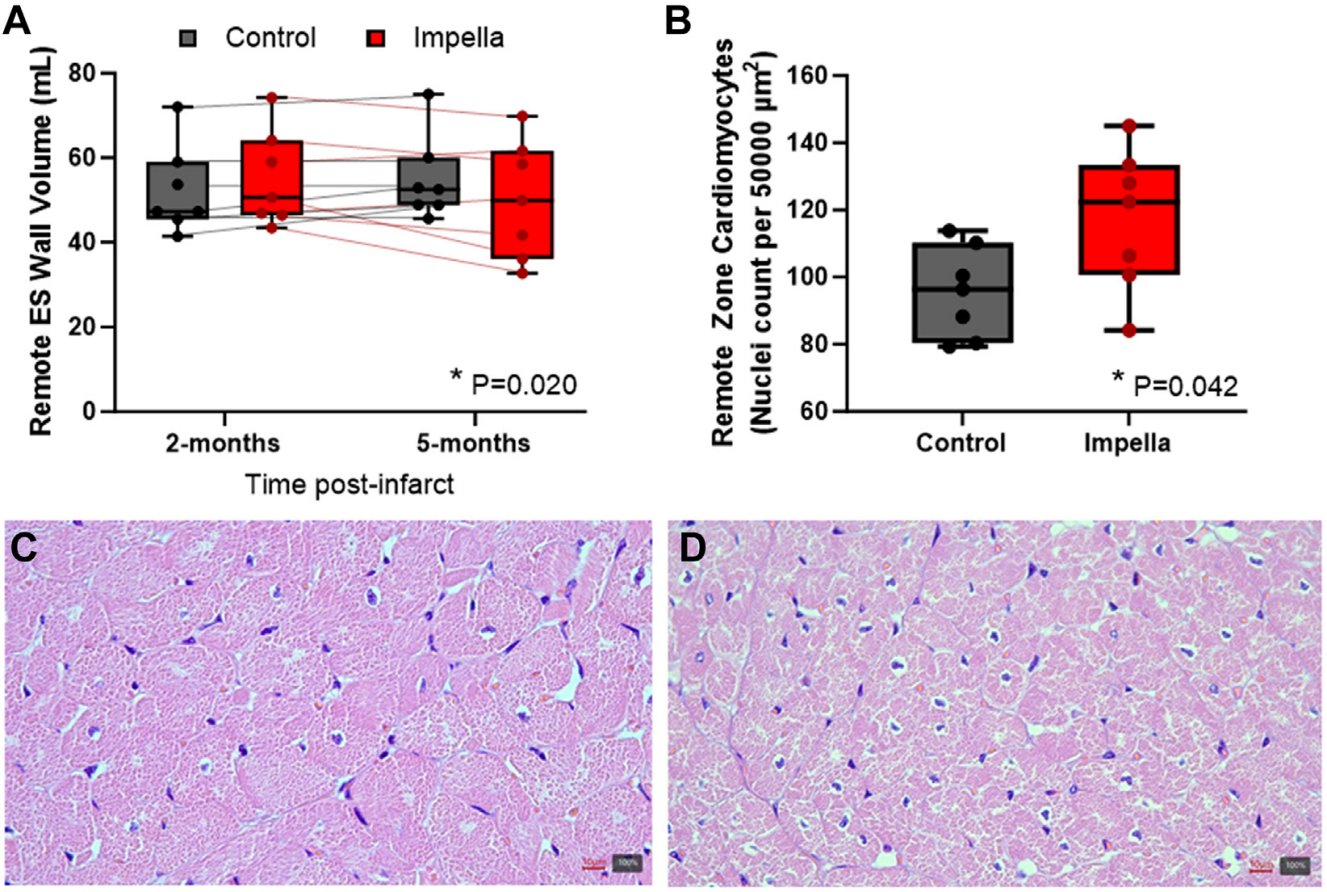
Transvalvular Unloading Reduces Cardiomyocyte Hypertrophy in Chronic Heart Failure Compensating hypertrophy occurs in control animals but not in Impella-supported animals, as demonstrated by (A) wall volume of the remote zone and (B) myocyte hypertrophy. Representative image of the remote myocardium of a (C) control animal and (D) a pump-supported animal at ×400 magnification. Note the relatively lower number of nuclei (both of cardiomyocytes and interstitial cells) in the control frame caused by cardiomyocyte hypertrophy, and the relatively higher number of nuclei (both of cardiomyocytes and interstitial cells) in the pump-supported frame caused by reverse remodeling. Box plots show the median with 25-75th percentiles (n = 7 control, 7 Impella) ∗*P <* 0.05, 2-way repeated measures analysis of variance time × treatment interaction (A), Student’s *t*-test (B).

To examine the hypothesis that Impella unloading in CHF has a favorable effect on remodeling of the remote zone postinfarct, CMR images were analyzed for regional LV function and structure. Impella support significantly improved regional EF and wall thickening of the remote zone in comparison to control (Figures 4A and 4B). Circumferential end-systolic strain also trended toward improvement in the Impella group, but was not statistically significant (Figure 4C). The end-systolic wall stress in the remote zone was significantly reduced after 3 months of Impella-mediated unloading, whereas control group worsened (Figures 4D and 4E). To investigate whether compensatory hypertrophy that occurs with CHF was halted or improved with Impella, the wall volume of the remote zone was analyzed. Failing control hearts had an increase in remote wall volume from 2 to 5 months, whereas Impella-supported hearts had a significant reduction (Figure 5A). Histology of the myocardium from the remote zone at the time of necropsy revealed that myocyte hypertrophy was also significantly decreased with Impella 5.5 support compared with control at 5 months (Figures 5B to 5D).

## Discussion

This study is the first to investigate whether the transvalvular mechanical circulatory support device, Impella 5.5, can be used to halt pathological remodeling and promote reverse remodeling in CHF. In an ischemic ovine model of CHF, 3 months of hemodynamic unloading was able to attenuate LV dilation with reduced LVESV and EDV in comparison to control. This result is in agreement with a previous report demonstrating reversal of CHF with an implantable ventricular assist device (VAD). Geens et al^8^ used an ovine model with HF development 6 weeks postinfarction. Their animals were 6 weeks unloaded and compared with no therapy with decreased LVEDV in the VAD group. With a longer duration of HF development (8 weeks) and longer duration of hemodynamic support (3 months), the present study further shows that regional myocardial structure and function is improved with transvalvular mechanical support. Improvements in regional EF, hypertrophy, and wall thickening of the remote zone demonstrate that, even in an ischemic HF model, reverse remodeling is possible by halting adverse remodeling and facilitating functional recovery in the remaining healthy tissue.

### Mechanism of reverse remodeling

In the present animal model with permanent ischemic damage, the driver of the observed reverse remodeling is the applied mechanical unloading, because it was the sole therapy used. Mechanical unloading affects several physiological mechanisms in the heart that may contribute to reverse remodeling: 1) reduction in wall stress; 2) reduction in energetic need of the myocardium; and 3) normalization of the neurohormonal environment for the heart.

The fall in LV volumes and pressures, caused by mechanical unloading, leads to a decrease in wall stress. Wall stress reduction can lead to changes in LV architecture that can be expressed by different parameters, such as reductions in LVESV, LVEDV, sphericity index, and mass index.^14^ The experience with LV reverse remodeling in resynchronization therapy has highlighted the importance of such a reduction in LVESV. The MADIT (Multicenter Automatic Defibrillator Implantation Trial) indicated that a reduction of LVESV is strongly associated with a lower risk for HF events.^15^ In follow-up studies, reducing LVESV proved to be the strongest parameter for long-term prognosis in terms of both the overall and cardiac-related survival. Patients with a remodeling of >35% of their LVESV had a 14% cumulative probability of all-cause mortality at the 6-year follow-up compared with 21% for patients with LVESV reductions ≤35%, and with 27% in patients receiving an implantable cardioverter-defibrillator only.^16^ The importance of geometric reversibility for VAD-promoted cardiac recovery was reported as well by Dandel et al.^17^ They showed in their long-term follow-up that a pre-explant LVEF >45% (a weaning requirement) revealed a positive predictive value for postexplant long-term stability of 79%, whereas its combination with a normal pre-explant LV end-diastolic dimension improved the predictive value up to 92%.^17^ In the current study, LVESV and LVEDV were both significantly reduced after 3 months of Impella unloading in addition to a reduction in myocyte-level hypertrophy. These data together suggest that 3 months of LV unloading with Impella 5.5 led to reductions in LV wall stress that resulted in LV architecture changes.

It is unclear if a normalization of the neurohormonal milieu by mechanical support contributed to the observed changes in this animal model. The relatively short time of treatment is an argument in favor of the mechanical pathway. Nevertheless, medical neurohormonal blockade has been shown to be a beneficial adjunctive therapy in LVAD patients, associated with both higher long-term survival and quality of life^18^ and is part of GDMT. In clinical scenarios, GDMT has been shown to promote reverse remodeling and stabilize the clinical CHF syndrome.^18^ The combination of GDMT with unloading therapy is important to maintain an optimized milieu for the heart in case of myocardial recovery and withdrawal of the pump. The durability of the unloading-mediated improvement in this animal experiment was not explored. However, the reduced wall stress observed after pump withdrawal is an encouraging sign of sustained impact of unloading. Centers reporting LVAD decommissioning after true myocardial recovery indicate encouraging results in the long-term follow-up of these patients.^5^^,^^6^^,^^19^^,^^20^

### Reversal from CHF

Results from the current study demonstrated that a less-invasive mechanical circulatory support device such as Impella 5.5 could provide sufficient degree of ventricular unloading to promote reverse remodeling without damaging the native heart. The current study focused on assessing the structural and functional recovery of the LV in response to unloading; however, recovery from CHF involves remodeling on the molecular level as well, and this should be explored in future studies.

For these findings to be translated into the clinical space, one of the main challenges is identifying the optimal timing and patient population to intervene for reverse remodeling. The University of Utah group^5^ identified younger age, nonischemic cardiomyopathy, recent diagnosis, absence of an implantable cardioverter-defibrillator, low creatinine levels, and smaller LV end-diastolic dimension as independent predictors of potential for reverse remodeling and created the I-CARS score based on these parameters. Biomarkers could be beneficial to guide the individual decision making and indicate the potential of unloading in CHF. Unfortunately, today, there is minimal evidence of reliable predictive values of specific biomarkers that can be used to indicate reverse remodeling and guide possible LVAD explant, and this is ground for future research.

In this study, the finding that, despite variability in cardiac remodeling, all mechanically supported animals showed a reversal of the remodeling process is promising. In addition, the animal model used is ischemic in nature, and the authors postulate that the results presented here may translate to the setting of nonischemic cardiomyopathy. Given the reported clinical experiences, decision making of potential for true myocardial recovery will have to consider not only the degree of geometric and functional reverse remodeling but also the etiology and duration of the underlying heart disease.^20^

The use of Impella 5.5 in CHF patients does entail the potential side effects of the therapy, such as potential bleeding complications and, in contrast to the use of implantable devices, the patient will not be able to leave the hospital.

### Study limitations

This study has several limitations, primarily in the ability of an animal model to fully mirror the clinical scenario. First, sheep with HF were only unloaded for 3 months. Recovery studies in durable LVAD populations suggest that on average a period of 6 months is required for mechanical unloading to facilitate reverse remodeling and recovery to explant. Further, device flow was not optimized for recovery, because pumps were set to run at maximum speed without suction. Third, the measurements of myocardial function were limited to baseline observations at time of assessment. Full understanding of recovery of function could not be tested in an exercise challenge. Last, the durability of Impella-mediated benefits postexplant was not investigated.

## Conclusions

Our findings suggest that hemodynamic unloading with Impella devices may offer CHF patients a therapy with the potential for recovery and remission. As described here, 3 months of unloading halted the progression of remodeling and revealed significant indications of reverse remodeling and functional improvement in an ischemic CHF model. Future studies should investigate whether optimization of Impella therapy, including duration of support, speed modulation and medical therapy, can further optimize reverse remodeling in ischemic CHF.

Perspectives **COMPETENCY IN MEDICAL KNOWLEDGE:** Durable VAD studies provided evidence that mechanical unloading of CHF can lead to reverse remodeling exhibited by reductions in end-systolic and -diastolic volumes and reduction of cellular hypertrophy. It can lead as well to remission with improvement of cardiac function. We investigated, for the first time, if this effect could be achieved with transvalvular mechanical support with Impella. In a model of ischemic cardiomyopathy, 3 months of unloading with Impella did lead to reverse remodeling and improvement of contractile function in the remote zone of the heart. The reduction in ventricular wall stress with Impella may contribute to the mechanism of reverse remodeling.

**TRANSLATIONAL OUTLOOK:** The present study enhances understanding of the mechanism of reversed remodeling in CHF, broadening the therapeutic options for recovery. Support with a less-invasive transvalvular pump appears to be an effective and promising strategy in CHF in addition to established medical therapy. The optimal moment of intervention and optimal duration of support is uninvestigated at this moment, and it remains a challenge to determine this in the trajectory of each individual CHF patient.

## Funding Support and Author Disclosures

Funding for this work was provided by a research grant from Abiomed, Inc. Dr Meyns has received institutional research grants from Abiomed. Drs Parks and Bennek Schoepping are employees of Abiomed. All other authors have reported that they have no relationships relevant to the contents of this paper to disclose.
